# Historical Assessment, Practical Management, and Future Recommendations for Abnormal Amniotic Fluid Volumes

**DOI:** 10.3390/jcm13164702

**Published:** 2024-08-10

**Authors:** Julie R. Whittington, Suneet P. Chauhan, Michael P. Wendel, Taylor L. Ghahremani, Megan E. Pagan, Meagen M. Carter, Everett F. Magann

**Affiliations:** 1 Department of Obstetrics and Gynecology, Navy Medicine Readiness and Training Command Portsmouth, Portsmouth, VA 23708, USA; julie.whittington09@gmail.com (J.R.W.);; 2 Department of Obstetrics, Gynecology and Reproductive Sciences, McGovern Medical School, The University of Texas Health Science Center at Houston (UTHealth), Houston, TX 77030, USA; 3 Department of Obstetrics & Gynecology, University of Arkansas for Medical Sciences, 4301 W. Markham St. Slot 518, Little Rock, AR 72205, USA; 4 Department of Obstetrics and Gynecology, Darnall Army Medical Center, Fort Hood, TX 76500, USA; 5 Department of Obstetrics and Gynecology, Virginia Tech Carillion, Roanoke, VA 24016, USA

**Keywords:** amniotic fluid volume, oligohydramnios, polyhydramnios, perinatal pregnancy outcome

## Abstract

**Objective:** The purpose of this review is to examine the evidence that defines normal and abnormal amniotic fluid volumes (AFVs) and current recommendations on the management of abnormalities of AFV. **Methods:** The studies establishing normal actual AFVs and the ultrasound estimates used to identify normal and abnormal AFVs were evaluated. Recommendations from national and international organizations were reviewed for guidance on the definitions and management of abnormal AFVs. **Results:** A timeline of the development of the thresholds that define abnormal AFVs was created. Recommendations from 13 national and international guidelines were identified, but the definitions and management recommendations for abnormal AFVs varied considerably between groups. An algorithm for the management of oligohydramnios and polyhydramnios was developed. Knowledge gaps and the structure of future studies were examined. **Conclusions:** Assessment of AFV is performed multiple times per day in antenatal clinics and hospitals. Current recommendations on defining and managing abnormal AFVs differ between national and international organizations. We have proposed algorithms to assist in the management of abnormal AFVs until further studies can be undertaken.

## 1. Introduction

Amniotic fluid is a multifunctional medium surrounding the fetus. It purportedly averts umbilical cord compression and fetal trauma and has bacteriostatic properties [1,2]. It provides nutrients and creates a space for fetal movement, allowing for neuromusculoskeletal maturation and prevention of pressure injuries [3]. Amniotic fluid is essential for fetal lung growth and gastrointestinal tract development. Sonographic estimation of amniotic fluid volumes is used clinically as a component to assess fetal well-being [4,5]. Abnormalities of amniotic fluid—putatively due to physiologic dysfunction in the triad of maternal, fetal, and placental unit [3]—are associated with adverse perinatal outcomes [6]. Ultrasound offers a pragmatic, non-invasive manner of assessing amniotic fluid. Sonographic estimation of amniotic fluid volumes has been extensively studied [5,7,8,9,10,11,12,13,14,15,16,17,18,19,20,21,22,23,24,25]. Researchers have studied the optimal way to measure amniotic fluid, how to identify abnormal fluid volumes, what the threshold to define abnormality is, and how to manage pregnancies with abnormal amniotic fluid measurements [22,26,27,28,29,30,31].

This review article aims to provide an overview of how abnormal values were established, how we approach amniotic fluid abnormalities, and review guidelines from professional organizations worldwide. This review includes the management of oligohydramnios and polyhydramnios in singletons. We also aim to highlight knowledge gaps that exist with respect to management of pregnancies with abnormal fluid measurements.

## 2. How Were Normal Amniotic Fluid Volumes Established?

Before the 1960s, there were very few studies on normal amniotic fluid volumes, as methods that could accurately and reliably measure fluid were limited. Dieckman and Davis first developed dye dilution techniques in the 1930s. With the application of ultrasound to obstetrics, a non-invasive assessment of amniotic fluid could be performed. First introduced in the literature in 1958, Ian Donald, John McVicar, and Tom Brown published the first paper to feature fetal images in *The Lancet*, entitled “The investigation of abdominal masses by pulsed ultrasound” [32,33]. In the 1970s and 1980s, the application of ultrasound in obstetrics began expanding dramatically. Images of the developing fetus could be taken in real-time, and dimensions of amniotic fluid pockets could be measured. In 1979, Hobbins et al. published one of the first papers showing the possibility of prenatal diagnosis of several congenital anomalies [34]. Also in 1979, Manning et al. first defined normal amniotic fluid and presented it at a meeting of the Society of Gynecologic Investigation; they subsequently published their findings on the development of a fetal biophysical profile in 1980 [35]. A year later, Manning described the association of low amniotic fluid on sonographic assessment and fetal growth restriction [36].

**Figure 1:**
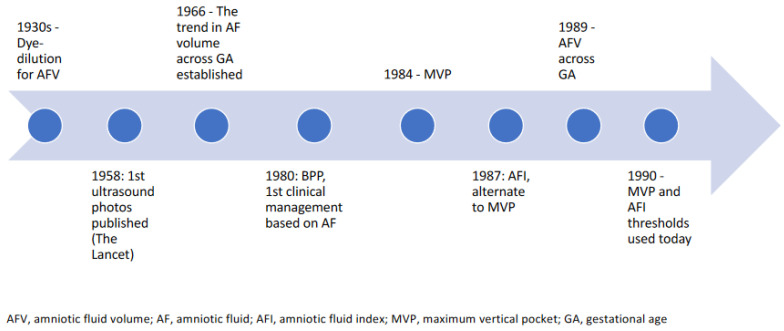
Timeline of development of amniotic fluid thresholds.

In 1984, Chamberlain introduced the maximal vertical pocket (MVP) [17]. The data were derived from ultrasound measurements on 7562 high-risk patients referred for biophysical profile testing. Using data from the BPP fluid measurements, they defined normal qualitative amniotic fluid volume (AFV) as >2 cm and <8 cm, increased AFV as ≥8 cm, decreased AVF as <1 cm, and marginal AFV as a pocket ≥1 cm and ≤2 cm. Two papers reported an association between decreased and increased qualitative AFV and adverse perinatal outcomes including perinatal death, congenital anomalies, and, in the case of decreased fluid, fetal growth restriction [16,17]. From July to September 1987, Phelan and Rutherford published studies on their experience with the AFI. In the final paper of that series, they established the normal values for the AFI in four groups: very low (0–5.0 cm), low (5.1–8.0), normal (8.1–18.0), and high (>18 cm) [13,14,19]. This was derived from the original study of 353 high-risk pregnancies between 36–42 weeks referred for fetal surveillance. In the final paper of this series, Rutherford et al. found that an AFI in the very low group (≤5.0 cm) was statistically associated with nonreactive NST, meconium, cesarean section for fetal distress, and Apgar score < 7 at 1 and 5 min. This became the basis of the lower limit of the AFI [13,19]. Moore and Cayle published a study that found the 97.5th percentile of fluid corresponded to an AFI of 24 cm, and ACOG subsequently adopted this as the upper limit of normal [7]. The 2.5th percentile, the threshold for oligohydramnios, roughly equated to an AFI of 7 cm [7]. This was not adopted, and an AFI of 5 cm remains the recommended lower limit of normal amniotic fluid volume [37]. These static cutoffs, while easy to apply throughout pregnancy, do not consider the regular amniotic fluid changes during gestation. In 1989, Brace and Wolf published a study demonstrating that amniotic fluid volumes change throughout gestation [38]. Later, a study by Magann et al. calculated the 5th and 95th percentiles of the AFI and SDP at each week of gestation. At each week of gestation from Weeks 14 to 41, 50 patients were examined, for a total of 1400 patients with normal pregnancies. Linear regression with logarithmic transformation ultimately showed that AFI corresponded to the original AFV chart plotted by Brace and Wolf, while MVP remained consistent throughout pregnancy [31]. Table S1 compares studies used to define normal sonographic amniotic fluid values. We also present a timeline of the history of the development of amniotic fluid thresholds (Figure 1).

## 3. Clinical Implications of Abnormal Fluid Volumes

### 3.1. Adverse Outcomes Associated with Oligohydramnios

The most common adverse outcome of oligohydramnios appears to be a result of iatrogenic prematurity [39]. A meta-analysis of 18 articles and over 10,000 parturients by Chauhan et al. reported that an AFI ≤ 5.0 cm, compared to 5.0 cm, was associated with an increased risk of cesarean delivery for fetal distress (RR 1.69; 95% CI 1.12–2.57) and an Apgar score of less than 7 at 5 min (RR 1.78; 95% CI 1.18–2.67) [5]. The authors remarked that they found the use of AFI problematic as they found a wide incidence of oligohydramnios in the included studies, from 3–40%. This wide variation in incidence reflects that numerous factors can affect AFI and therefore its impact and reliability for use in clinical decision-making.

A systematic review and meta-analysis of oligohydramnios by Rabie et al., which included a total of 15 trials and 8067 high-risk people and 27,526 low-risk people, found that uncomplicated singleton pregnancies with oligohydramnios were more likely to have an emergency cesarean delivery for fetal distress (RR, 2.16; 95% CI, 1.64–2.85), admission to the neonatal intensive care unit (NICU) (RR, 1.71; 95% CI, 1.20–2.42), and meconium-aspiration syndrome (MAS) (RR, 2.83; 95% CI, 1.38–5.77) [40]. Patients with oligohydramnios and other comorbid conditions were more likely to have an infant with low birth weight (RR, 2.35; 95% CI, 1.27–4.34), but there was no increase in fetal distress, NICU admission, or MAS. The study could not determine if there was an increased risk of stillbirth due to the low incidence of stillbirth in the included studies [40]. In comparing these two meta-analyses, there is a commonality of increased risk of cesarean delivery for fetal distress. However, the meta-analysis by Rabie et al. isolated the outcomes based on an otherwise low-risk pregnancy versus a high-risk pregnancy, indicating the difference in outcomes between the two. On the contrary, a cohort study by Zhang et al. of the multi-center clinical trial of RADIUS found similar perinatal outcomes in complicated and uncomplicated pregnancies with oligohydramnios compared to those with normal AFI [41].

In a case–control study of preterm oligohydramnios from Israel, oligohydramnios has been associated with a subsequent diagnosis of fetal growth restriction [39]. Melamed et al. reported that isolated oligohydramnios had an increased risk of adverse pregnancy outcomes related to iatrogenic prematurity [39]. They found no other increased risk of other adverse outcomes. However, the study was not powered to detect stillbirth. The study did find an increased rate of fetal growth restriction (estimated fetal weight <10th percentile using the local reference group) diagnosed after the identification of oligohydramnios in patients who were managed expectantly but not an increased rate of being small for gestational age (birthweight <10th percentile) or other morbidities [39].

In summary, oligohydramnios in singletons confers an increased risk of certain adverse perinatal outcomes, although the data on whether other perinatal comorbidities play a role is conflicting. The authors of this review continue to advocate for the use of maximum vertical pocket over amniotic fluid index for diagnosis of oligohydramnios.

### 3.2. Adverse Outcomes Associated with Polyhydramnios

It is estimated that polyhydramnios complicates up to 1% of pregnancies; approximately half are idiopathic [42]. Polyhydramnios is most commonly associated with maternal diabetes and fetal anomalies. Other causes of polyhydramnios include high fetal cardiac output, as in fetal anemia (due to alloimmunization or parvovirus B19 infection). When no identifiable etiology is ascertained, it is considered idiopathic polyhydramnios. Ten percent or more of pregnancies with idiopathic polyhydramnios will have a structural or genetic abnormality diagnosed after birth [42,43]. Idiopathic polyhydramnios has been found to be associated with an increased risk of fetal macrosomia (≥4000 g) in several studies [44,45,46,47]. Odibo et al. found that 20.5% of pregnancies with persistent idiopathic polyhydramnios had fetal macrosomia [44]. It is also associated with an increased rate of cesarean delivery, with an incidence of up to 44% [44,45,47]. There are inconsistent reports of the risk of perinatal mortality associated with idiopathic polyhydramnios. Several studies have found no increased risk of perinatal mortality (Khan, Wiegand, Panting-Kemp) [45,47,48]. However, in a study by Pri-Paz, the intrauterine fetal demise rate was increased (1.2%), and Biggio et al. found a 3.7% perinatal mortality rate [46,49]. Luo et al. published a study of idiopathic polyhydramnios (anomalies excluded) that included over 100,000 pregnancies [50]. Although the incidence of fetal death was only 0.7%, the sample size was large, and they detected a 17-fold increased rate of fetal death in mild idiopathic polyhydramnios. The rate was dramatically increased for moderate to severe polyhydramnios, with an 85-fold increased rate of fetal death [50].

A systematic review and meta-analysis in 2022 of singleton pregnancies with idiopathic polyhydramnios showed an odds ratio of 8.68 (95% CI: 2.91, 25.87) for neonatal death in the polyhydramnios group, and an odds ratio of 7.64 (95% CI: 2.50, 23.38) for intrauterine fetal demise [20]. Within the same study, overall pooled results revealed that the polyhydramnios group had greater odds of NICU admission [OR = 1.94; 95% CI: 1.45, 2.59; I^2^ = 52%] [20]. Most of the studies in this meta-analysis did not show an association with polyhydramnios and 5 min APGAR score < 7, but the pooled analysis showed an odds ratio of 2.21 (95% CI: 1.34, 3.62; I^2^ = 33%). There was also a higher rate of cesarean delivery in the polyhydramnios group (OR of 2.31 [95% CI: 1.79, 2.9 9; I^2^ = 80%]), higher rates of macrosomia (OR = 2.93 with 95% CI: 2.39 to 3.59), and higher odds of malpresentation (OR 2.73, 95% CI: 2.06, 3.61) [20]. Although this meta-analysis showed an increased risk of adverse outcomes, the majority of the included studies were retrospective, and the risk of bias was affected by this. However, with the large sample size, it may be more reflective of the risk of perinatal mortality because idiopathic polyhydramnios complicates a very small number of pregnancies; a considerable sample size is needed to adequately power a study to detect an increased rate of perinatal mortality. The studies by Luo et al. and the recent meta-analysis have a large sample size and show an increased rate of perinatal morality. However, data about antenatal surveillance was lacking [20,50].

The literature on idiopathic polyhydramnios indicates an increased risk of macrosomia and cesarean delivery, but there are mixed data on rates of fetal demise. However, based on the most recent systematic review and meta-analysis, there does appear to be an increased risk of perinatal mortality.

## 4. Guideline Review—Evidence Acquisition

Considering the multitude of publications on oligohydramnios and polyhydramnios (over 6800 in PubMed), a review of guidelines established by national organizations is useful. We searched the websites of the American College of Obstetricians and Gynecologists (ACOG), the American Institute of Ultrasound in Medicine (AIUM), the French College of Gynecologists and Obstetricians (CNGOF), the Fetal Medicine Foundation, the International Federation of Obstetrics and Gynecology (FIGO), the International Society of Ultrasound in Obstetrics and Gynecology (ISUOG), the Japanese Association of Obstetricians and Gynecologists (JAOG), the National Institute for Health and Care Excellence (NICE), the National Health Service of Glasgow and Clyde (NHSGGC), the Royal Australian New Zealand College of Obstetricians and Gynecologists (RANZCOG), the Royal College of Obstetricians and Gynaecologists (RCOG), the Society for Maternal-Fetal Medicine (SMFM), and the Society of Obstetricians and Gynaecologists of Canada (SOGC) [51,52,53,54,55,56,57,58,59,60,61,62,63]. We specifically sought to review guidelines regarding the definition of abnormal amniotic fluid (oligohydramnios or polyhydramnios) and review management guidelines of assessment, antepartum surveillance, and delivery timing.

## 5. Discussion

### 5.1. How Do Professional Organizations and Societies Define Normal and Abnormal Amniotic Fluid Volumes?

There is variation in the definition of normal sonographic estimation of amniotic fluid between the 13 resources we identified. (Table S1) While eight references defined normal amniotic fluid, five references did not define what a normal amniotic fluid volume measurement is. References varied in the use of the deepest vertical pocket, the amniotic fluid index, or both. The terms deepest vertical pocket, maximum vertical pocket, single deepest pocket, and deepest pool are interchangeable, and each organization uses nomenclature of their choosing. Table S2 describes thresholds for normal and abnormal amniotic fluid.

One difference is that the SOGC designates the measured pocket’s horizontal dimension (2 × 1 cm and 8 × 1 cm) [64]. Notably, Manning et al. originally described a pocket greater than 1 cm in diameter in their introduction of the biophysical profile, then moved to a 1 × 1 cm pocket as sufficient and finally to a 2 cm pocket [35,36,65]. Another difference is that RANZCOG uses an amniotic fluid index of over 20 cm to define polyhydramnios [66]. This is understandable given that the range of AFI measurements used to define polyhydramnios is broad (18–25 cm) [7,13,67].

### 5.2. Management of Abnormal Amniotic Fluid Volumes

There are few data from various professional organizations and societies on managing amniotic fluid abnormalities (Tables S3 and S4). Of 13 organizations, 7 give recommendations for assessing oligohydramnios after diagnosis, and many share joint publications on ultrasound examination [54,68,69,70,71]. A detailed amniotic survey and evaluation for rupture of membranes are advised. The authors posit that maternal assessment should be incorporated into a standardized management algorithm, including medical comorbidities and evaluation for hypertensive disorders of pregnancy [40]. Only 3 of 13 organizations give recommendations for antenatal testing with oligohydramnios [69,72]. This may be because some of the referenced organizations are oriented toward diagnosing conditions and not necessarily offering recommendations for management. JAOG recommends assessment of fetal well-being but does not specify the frequency or type of testing [69]. ACOG and SMFM recommend initiating antenatal testing at diagnosis (if delivery would be considered) [72]. Regarding delivery timing for oligohydramnios, only 2 of 13 organizations give recommendations. ACOG and SMFM share a joint publication regarding recommendations for the timing of late-preterm and early-term deliveries and recommend delivery at 36 0/7–37 6/7 weeks or at diagnosis if diagnosed later [73]. JAOG advises consideration of amnioinfusion in labor for oligohydramnios to relieve cord compression [69].

Of 13 organizations, 8 do not give recommendations for evaluation of polyhydramnios; 4 of 13 organizations recommend a detailed amniotic survey with evaluation for causes of polyhydramnios, including infection, fetal abnormalities, hydrops fetalis, and skeletal dysplasias [42,54,59,68]. SMFM includes the importance of assessing fetal movement, as neurologic disorders can affect fetal swallowing [42]. JAOG broadly recommends investigating the cause of polyhydramnios [69]. Antenatal testing and evaluation is not necessarily indicated for mild polyhydramnios; however, the authors consider antenatal testing due to the recent meta-analysis by Pagan et al. that indicates increased risk of perinatal mortality [20]. ACOG recommends antenatal testing for moderate to severe polyhydramnios. With respect to delivery, 4 of 13 organizations offer recommendations. Three of these four organizations give delivery timing recommendations [42,54,73]. ACOG and SMFM note that delivery for mild polyhydramnios should not be recommended before 39 weeks of gestation and recommend individualization of delivery timing for moderate to severe polyhydramnios [42,73]. The Fetal Medicine Foundation recommends delivery at 38 of weeks gestation for severe polyhydramnios [54]. They also give recommendations for location of delivery depending on whether a fetal abnormality is expected [74]. RANZCOG recommends that intrapartum cardiotocography be performed in cases of polyhydramnios [66].

**Figure 2:**
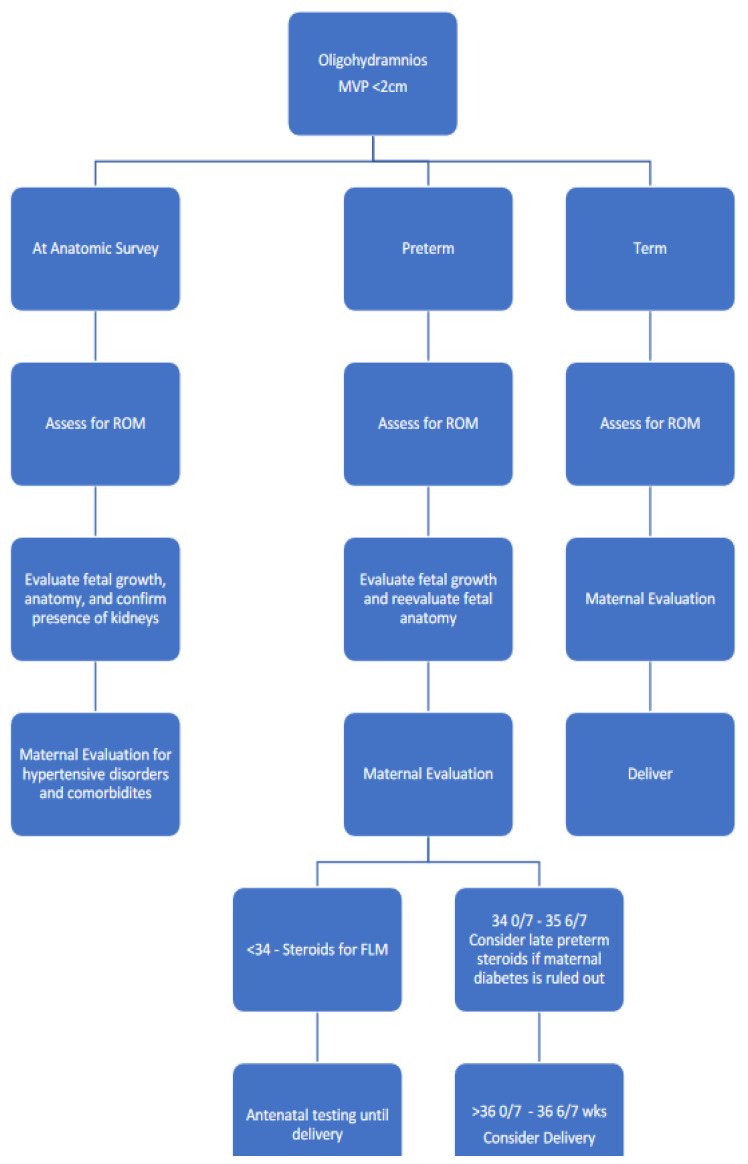
Recommended management of oligohydramnios per gestational week. ROM, rupture of membrane; FLM, fetal lung maturity.

**Figure 3:**
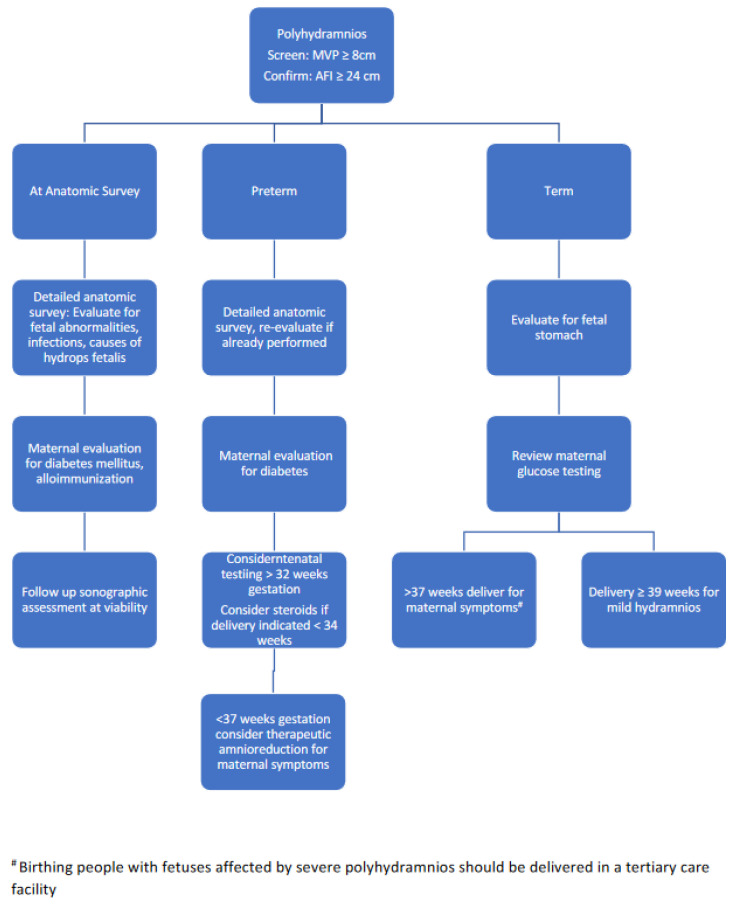
Recommended management of polyhydramnios per gestational week.

Based upon the body of literature, organizational recommendations, and our clinical experience, we offer recommendations on the management of sonographic amniotic fluid abnormalities (Figure 2 and Figure 3).

### 5.3. Knowledge Gaps

Knowledge gaps regarding abnormalities of amniotic fluid abound. This is self-evident in the summary of the 13 guidelines and the apparent lack of consensus among them vis-à-vis whether to use MVP or AFI, the threshold of abnormalities, and the management schema once oligo- or polyhydramnios is diagnosed. Herein, we describe some of the knowledge gaps and potential study design to address them.

To begin with, a descriptive multicenter epidemiological study on the rate of abnormalities of amniotic fluid and their associated adverse outcome to the maternal–newborn dyad is warranted. Though Owen J et al. do provide a contemporary standard for the US for amniotic fluid estimation that uses the SDP and AFI, it is limited to low-risk pregnancies and is not linked with peripartum outcomes [75]. The corollaries for the observational study are that high-risk pregnancies (e.g., individuals with diabetes or hypertensive disorder) have a separate nomogram for amniotic fluid and that their findings may not apply to individuals abroad [12,76]. Importantly, without data on peripartum outcomes, neither clinicians nor researchers are able to discern if extremes of dispersion (i.e., less than 3rd or greater than 97th percentile) are linked with adverse outcomes.

A StaRI (Standards for Reporting Implementation Studies)-compliant pre- and post-interventional trial utilizing AFI and then MVP (or vice versa) to diagnose and manage oligo- and polyhydramnios would address which method of assessing amniotic fluid is associated with fewer iatrogenic prematurity and adverse outcomes [42,77]. ACOG and SMFM guidelines recommend use of MVP to diagnose and manage oligohydramnios, but SMFM notes that MVP potentially over-diagnosing polyhydramnios is a clinical conundrum that needs to be addressed [42,72]. While the association of adverse outcomes to newborns with oligohydramnios or polyhydramnios is known, one method of assessing AF for all individuals is preferable [5,20,40].

Reports on amniotic fluid abnormalities often have an admixture of low- and high-risk pregnancies. Presumably, the likelihood of adverse outcomes of oligohydramnios with hypertensive disorder or fetal growth restriction is different than among otherwise “low-risk” individual. Similarly, hydramnios with and without diabetes may have different implications. But the relative magnitude of differences is missing and should be addressed in a STARD (Studies of Diagnostic Accuracy)-compliant observational study [78]. A study with these standards will not only assist with counselling individuals but also with policymaking and study design for interventional trials.

A CONSORT (Consolidated Standards of Reporting Trials)-compliant individually consented randomized clinical trial to decrease the currently known rates of adverse outcomes would be difficult to accomplish but needs to be performed [79]. The impetus for such a trial is that amniotic fluid is assessed with every sonographic examination. Most individuals have multiple ultrasound examinations during pregnancy, and abnormalities of fluid may affect upward of 10% of births. Thus, considering that there were ~3.66 million births in 2021 in the US, we estimate that annually, there are approximately 366,000 cases of oligo- or polyhydramnios [80]. Previously, the National Institute of Child Health and Human Development funded multi-center prospective longitudinal data collection to develop contemporary fetal growth and amniotic fluid standards for four self-identified US racial/ethnic groups [75,81]. A similar funding opportunity is needed if the adverse outcomes (e.g., stillbirth, cesarean delivery, low Apgar score) linked with aberrant amniotic fluid are to be reduced.

Abnormalities of amniotic fluid before 34 weeks and how to manage them have not been sufficiently addressed in a large multi-center study. For oligohydramnios, the options include admission, hydration, possible antenatal corticosteroids, and repeat ultrasound examination. However, if the abnormalities persist, there is a variance in clinical practice on whether to manage them as an in- or outpatient. Trials addressing this issue could minimize the variance and improve outcomes. Lastly, the long-term outcomes of newborns with history of oligo- and polyhydramnios are inadequately reported, as is the rate of reoccurrence of abnormal amniotic fluid.

Amniotic fluid assessment is an important part of evaluation of the maternal-fetal-placental unit and guides evaluation and management of the pregnancy. There are currently no robust international recommendations on the diagnosis and management of amniotic fluid abnormalities. We offer a practical algorithm for managing oligohydramnios and polyhydramnios that may help clinicians with evaluation and decision-making. Finally, we highlight the fact that despite the large body of work in amniotic fluid, knowledge gaps persist, and more work needs to be done.

## Data Availability

Not applicable.

## Supplementary Materials

The following supporting information can be downloaded at: https://www.mdpi.com/article/10.3390/jcm13164702/s1, Table S1: Studies establishing normal amniotic fluid volumes; Table S2: Thresholds for normal and abnormal amniotic fluid; Table S3: Management recommendations by professional organizations and ultrasound journals for oligohydramnios; Table S4: Management recommendations by professional organizations and ultrasound journals for polyhydramnios.
