# Supplementary material for: Historical Assessment, Practical Management, and Future Recommendations for Abnormal Amniotic Fluid Volumes
Source: J Clin Med. 2024 Aug 10;13(16):4702. doi: 10.3390/jcm13164702 (PMC11355879; doi:10.3390/jcm13164702)
Supplement: Supplementary file 1 [file jcm-13-04702-s001.zip › jcm-3134886-supplementary.pdf]

Table S1. Studies establishing normal amniotic fluid volumes.

Author	N	Exam by RDMS / MD	Patient Population	Classification of amniotic fluid measurement
Manning (1980)	216	MD	Antepartum high-risk patients referred for BPP	"Fluid evident throughout the uterine cavity. Largest pocket of fluid greater than 1cm in the vertical diameter."
Chamberlain (1984)	7562	MD	Antepartum high-risk patients referred for BPP	Normal >2cm and <8cm, increased ≥8cm, decreased <1cm, and marginal ≥1cm and ≤2cm
Phelan J (1987)	353	MD	Antepartum high-risk patients referred for antenatal testing	Very low 0-5.0cm, low 5.1-8.0, normal 8.1-18.0, and high >18cm
Moore (1990)	791	RDMS	Antepartum normal pregnancies	2.5th percentile <7.0cm, 97.5th percentile >24.0cm
Magann (2000)	1400	MD	Antepartum normal pregnancies	Variable, calculated 5th and 95th percentiles for each week of gestation
Owen (2019)	1719	Credentialed Sonographer	Antepartum low-risk pregnancies	Variable, calculated at the 3rd and 97th percentiles for each week of gestation

Table S2. Thresholds for normal and abnormal amniotic fluid.

RESOURCE	COUNTRY	NORMAL AF ^	OLIGOHYDRAMNIOS ^	POLYHYDRAMNIOS^
ACOG (2021, 2022)	United States	DVP ≥ 2 – ≤ 8	DVP < 2	Mild: DVP 8 – 11 or AFI 24 – 29.9<br>Moderate: DVP 12 – 15 or AFI 30 – 34.9<br>Severe: DVP ≥ 16 or AFI ≥ 35
AIUM (2019)	United States	DVP ≥ 2 – DVP < 8<br>AFI > 5 – AFI < 24	DVP < 2 or AFI ≤ 5	DVP ≥ 8 or AFI ≥ 24
CNGOF	France	Not specified	Not specified	Not specified
FIGO	United Kingdom	Not specified	Not specified	Not specified
FMF	United Kingdom	DVP ≥ 2 - <8	DVP < 2 or AFI < 5	Mild: DVP 8-11<br>Moderate: DVP 12-15<br>Severe: DVP ≥ 16
ISUOG (2022)	United Kingdom	DVP >2 - ≤8	DVP ≤ 2 or AFI <5-6	DVP > 8
JAOG (2022)	Japan	Amniotic Pocket (AP) ≥2 - ≤ 8	AP <2 or AFI <5	AP >8 or AFI >25
NICE	United Kingdom	Not specified	Not specified	Not specified
NHSGGC	United Kingdom	Deepest pool ≥ 2 - ≤ 8	Deepest pool < 2	Deepest pool > 8
RANZCOG (2019)	Australia	MVP ≥ 2 - ≤ 8<br>AFI ≥ 5 - 20	MVP < 2 or AFI < 5	MVP > 8 or AFI > 20[#]
RCOG	United Kingdom	Not specified	SDVP < 2	Not specified
SMFM * (2018)	United States	-	-	Mild DVP 8 – 11 or AFI 24 – 29.9<br>Moderate DVP 12 – 15 or AFI 30 – 34.9<br>Severe DVP ≥ 16 of AFI ≥ 35
SOGC	Canada	SDP ≥ "2 X 1" - ≤ "8 X 1"	SDP < "2 X 1"	SDP > "8 X 1"

ACOG = American College of Obstetricians and Gynecologists; AIUM = American Institute of Ultrasound in Medicine; CNGOF = The French College of Obstetricians and Gynecologists; FIGO = International Federation of Gynecology and Obstetrics; FMF = Fetal Medicine Foundation; ISUOG = International Society of Ultrasound in Obstetrics and Gynecology; JAOG = Japan Association of

Obstetricians and Gynecologists; NICE = National Institute for Health and Care excellence; NHSGGC = National Health Service of Glasgow and Clyde; RANZCOG = Royal Australian and New Zealand College of Obstetricians and Gynaecologists; RCOG = Royal College of Obstetricians and Gynaecologists: SMFM = Society of Maternal Fetal Medicine; SOGC = Society of Obstetricians and Gynaecologists.

^ all measurements are in cm.

* SMFM guideline is for polyhydramnios only.

#Or as defined by local guidelines.

Table S3. Management recommendations by professional organizations and ultrasound journals for oligohydramnios.

RESOURCE	Baseline assessment upon diagnosis	Antenatal testing	Delivery timing and considerations
ACOG	Detailed anatomic survey	At diagnosis, if delivery would be considered	36 0/7 – 37 6/7 weeks or at diagnosis if diagnosed later
AIUM (2019)	Detailed anatomic survey	No recommendations	No recommendations
CNGOF	No recommendations	No recommendations	No recommendations
FIGO	No recommendations	No recommendations	No recommendations
FMF	Detailed ultrasound examination, consider invasive testing	No recommendations	No recommendations
ISUOG	Detailed ultrasound examination	No recommendations	No recommendations
JAOG (2020)	Evaluate urinary tract and evaluate for rupture of membranes	Assess fetal well-being	Intrapartum amnioinfusion can be considered to reduce cord compression
NICE	No recommendations	No recommendations	No recommendations
NHSGGC	Check fetal kidneys and bladder, evaluate for growth restriction and rupture of membranes	No recommendations	No recommendations
RANZCOG	No recommendations	No recommendations	No recommendations
RCOG	No recommendations	No recommendations	No recommendations
SMFM#	Detailed anatomic survey	At diagnosis, if delivery would be considered	36 0/7 – 37 6/7 weeks or at diagnosis if diagnosed later
SOGC	No recommendations	No recommendations	No recommendations

#SMFM-endorsed ACOG recommendations for outpatient antenatal surveillance and medically indicated late-preterm and early-term deliveries.

Table S4. Management recommendations by professional organizations and ultrasound journals for polyhydramnios.

RESOURCE	Baseline assessment upon diagnosis	Antenatal testing recommendation	Delivery timing and considerations
ACOG (2021)	No Recommendations	Moderate to Severe at 32 0/7 – 34 0/7 weeks	Mild: 39 0/7 – 40 6/7 weeks if isolated<br>Moderate, severe: individualize
AIUM	No recommendations	No recommendations	No recommendations
CNGOF	No Recommendations	No Recommendations	No Recommendations
FIGO	No Recommendations	No Recommendations	No Recommendations
FMF	Detailed anatomic survey, invasive testing for fetal abnormalities, glucose tolerance test if macrosomia is present, torch titers if signs of infection	Ultrasounds every 1-3 weeks to monitor amniotic fluid volume, and cervical length	For fetal abnormalities: induction and delivery at 38 weeks at tertiary center with pediatric surgery<br>For fetal tumors of neck: cesarean and EXIT procedure<br>Severe: deliver at 38 weeks
ISUOG	Detailed anatomic survey, evaluate for diabetes, genetic disorders, multiple gestation, infections, metabolic disorders, isoimmunization	No Recommendations	No Recommendations
JAOG	Investigate the cause of the polyhydramnios	Assess fetal well-being by assessing fetal parameters, such as fetal movement, consider amnioreduction in women with large uterus or signs of premature labor	No Recommendations
NICE	No Recommendations	No Recommendations	No Recommendations
NHSGGC	Detailed anatomic survey looking for skeletal dysplasia, exclude gross fetal abnormalities, look for fetal stomach	No Recommendations	No Recommendations
RANZCOG	No Recommendations	No Recommendations	Intrapartum Cardiotocography is Recommended
RCOG	No Recommendations	No Recommendations	No Recommendations
SMFM * (2018)	Detailed anatomic survey to assess for abnormalities, fetal growth, fetal movement, placental abnormalities, diabetes screening	No data that antenatal testing reduces mortality	Mild: Not before 39 0/7 weeks<br>Severe: deliver at a tertiary care center due to risk of anomalies
SOGC	No Recommendations	No Recommendations	No Recommendations

EXIT—ex-utero intrapartum treatment.
